# The CRISPR-Cas toolkit for mosquito-borne virus surveillance: detection, tracing, and discovery

**DOI:** 10.3389/fcimb.2026.1873187

**Published:** 2026-06-18

**Authors:** Yang Wu, Huiling Cai, Qipeng Wu, Jie Wu, Jia Hu, Enjiong Huang, Zhongqi Li, Shaojun Liang, Xuefeng Hu, Jun Dai, Ruyan Liao

**Affiliations:** 1 Guangzhou Customs Technology Center, Guangzhou, China; 2 State Key Laboratory of Respiratory Disease, Guangzhou, China; 3 Fuzhou International Travel Healthcare Center, Fuzhou, China; 4 Jilin International Travel Healthcare Center, Tonghua, China; 5 Guangzhou International Travel Healthcare Center, Guangzhou, China; 6 Jiangsu International Travel Healthcare Center, Nanjing, China

**Keywords:** CRISPR-Cas, mosquito-borne viruses, outbreak phylodynamics, point-of-need diagnostics, targeted multiplex screening, variant-aware detection

## Abstract

Mosquito-borne virus surveillance increasingly requires rapid, distributed detection of co-circulating pathogens, serotypes, and lineages across clinical and vector-sampling sites. CRISPR-Cas platforms offer a programmable toolkit for this purpose, but their readiness differs substantially across surveillance functions. Here, we review CRISPR-Cas methods for mosquito-borne virus surveillance across detection, tracing, and discovery-supporting targeted screening. Detection is the most advanced application: selected Cas12- and Cas13-based assays for dengue, Zika, chikungunya, West Nile, Japanese encephalitis, and related mosquito-associated viruses report sub-hour workflows, portable readouts, and targeted serotype- or lineage-marker discrimination. However, performance remains assay-, target-, and sample-matrix-dependent, and validation in pooled mosquito samples and field settings is still limited. Tracing currently relies mainly on validated portable amplicon-sequencing workflows, whereas CRISPR-aided sample-preparation methods such as DASH, FLASH, RAPID-DASH, and Cas9-targeted enrichment remain transferable opportunities for host depletion or target enrichment rather than established mosquito-borne virus genomic-surveillance workflows. For discovery-oriented surveillance, multiplex CRISPR-Cas systems such as CARMEN can support targeted screening of known or near-neighbor viruses represented by predesigned crRNAs, while metagenomic next-generation sequencing remains necessary for divergent or previously unknown viruses. Across these functions, CRISPR-Cas programmability may accelerate parts of assay redesign, but practical retargeting still requires compatible amplification primers, effector-specific target constraints, cross-reactivity assessment, and analytical revalidation. Routine surveillance use will require integrated demonstrations with clinical and pooled-vector samples, comparison against established molecular and sequencing methods, cost validation, and regulatory evidence.

## 1 Introduction

The demand pattern facing arbovirus surveillance no longer matches its architecture. Over the past sixty years dengue has spread throughout the tropical world and now affects more than half of the world’s population (Messina et al., 2019) and the geographic footprints of dengue, chikungunya, Zika, West Nile virus (WNV) and Japanese encephalitis virus (JEV) continue to expand, including *Aedes albopictus*-driven expansion of dengue and chikungunya transmission risk into temperate Europe (Farooq et al., 2025). The first autochthonous WNV outbreaks in previously WNV-free European areas have been reported in recent years, accompanied by the genomic emergence of WNV lineage 2 sublineages (Koch et al., 2024; Vignjević et al., 2024). Ongoing JEV transmission in *Culex* vectors continues to drive demand for rapid clinical and vector-side detection, as exemplified by recent methodology development (You et al., 2024). Surveillance must now operate against multiple pathogens, multiple lineages and multiple geographies at once, with shorter response windows and a denser distribution of testing nodes than the centralized reverse-transcription quantitative PCR (RT-qPCR) architecture was built to support.

This gap is unevenly distributed. Latin America and the Caribbean carry the largest current arboviral burden, with rising dengue transmission potential and recent outbreak surges (Hartinger et al., 2024). In Africa the gap is one of underdetection rather than scale, where malaria diagnostic infrastructure dominates clinical workflows (Ateutchia Ngouanet et al., 2022) and dedicated molecular-surveillance studies routinely confirm arbovirus cases that conventional reporting systems miss (Shaibu et al., 2023). Temperate regions are now joining the affected geographies, with rising autochthonous-transmission risk across previously unexposed areas (Gizaw et al., 2024). The largest deficit sits in peripheral health systems.

These dynamics impose four requirements. Time from sample to actionable result must be measured in tens of minutes, not hours. Testing must extend from centralized laboratories out to peri-urban clinics, sentinel sites and entomological traps. Each reaction must accommodate multiple co-circulating serotypes, lineages and pathogens. And assay components must be adaptable on a days-to-weeks timescale, including guide RNAs and compatible amplification primers, faster than full redevelopment of standard amplicon panels (Huang et al., 2024). These requirements are difficult to meet with centralized RT-qPCR alone, particularly when surveillance must be distributed across peripheral clinical, sentinel, and vector-monitoring sites.

CRISPR-Cas systems developed over the past decade can address parts of this requirement set. Class 2 effectors (Cas12, Cas13, Cas12f) recognize their nucleic-acid target through a programmable CRISPR RNA (crRNA), which can be redesigned by solid-phase oligonucleotide synthesis. On recognition, their trans-cleavage activity provides a multi-turnover signal-amplification step that reaches attomolar analytical sensitivities comparable to those of RT-qPCR (Gootenberg et al., 2017; Chen et al., 2018; Harrington et al., 2018). Collateral cleavage places the time to result in tens of minutes. crRNA design is increasingly augmented by deep-learning optimization, which supports rapid in silico guide selection on the timescale of oligonucleotide synthesis turnaround (Huang et al., 2024). Orthogonal Cas effectors enable single-reaction multiplexing across four channels (Gootenberg et al., 2018), and the Combinatorial Arrayed Reactions for Multiplexed Evaluation of Nucleic acids (CARMEN) microwell-array architecture extends this to over 4,500 crRNA-target pairings on a single array (Ackerman et al., 2020). Lyophilized one-pot reagents extend assay shelf life under ambient conditions, as demonstrated for bacterial pathogens (Cao et al., 2025), while compatible smartphone-readable fluorescence and lateral-flow readouts move detection out of centralized laboratories to clinics, traps and sentinel sites (Arizti-Sanz et al., 2020).

The CRISPR-Cas literature on mosquito-borne virus surveillance is organized most usefully around three functions: detection, tracing, and discovery-oriented screening. Detection asks whether a specific mosquito-borne virus target is present and, where assays are designed for it, whether predefined serotype, genotype, or lineage markers can be discriminated. Several mature one-pot CRISPR-Cas platforms now address these questions: Specific High-sensitivity Enzymatic Reporter UnLOCKing (SHERLOCK), DNA Endonuclease-Targeted CRISPR Trans Reporter (DETECTR), One-HOur Low-cost Multipurpose highly Efficient System (HOLMES), Streamlined Highlighting of Infections to Navigate Epidemics (SHINE), and Extraction-Free One-pot recombinase-aided amplification (RAA)-CRISPR/Cas13a (EFORCA). Lineage- and serotype-specific assays now place this discrimination within the same one-pot reaction as the detection itself. Tracing picks up where detection ends, reconstructing transmission chains and outbreak phylodynamics from recovered pathogen sequences. The Zika in Brazil Real-time Analysis (ZiBRA)-style portable amplicon-sequencing template made this feasible at outbreak speed. Several CRISPR-aided sample-preparation methods now lower per-sample bioinformatic burden at the library-preparation step enough for peripheral sequencing nodes: Depletion of Abundant Sequences by Hybridization (DASH), Finding Low Abundance Sequences by Hybridization (FLASH), RAPID-DASH and Cas9-targeted nanopore enrichment. Discovery-oriented surveillance targets the same vector pools through targeted multiplex screening of known or near-neighbor viruses represented by predesigned crRNAs. Multiplex CARMEN-Cas13 architectures provide the targeted breadth, paired with metagenomic next-generation sequencing (NGS) as the unbiased fallback. The biological substrate is the mosquito virome, in which insect-specific viruses (ISVs) shape the vector competence that arboviruses must navigate (Wu et al., 2025).

These observations motivate organizing the CRISPR-Cas literature around three surveillance functions: detection, tracing, and discovery. The primary focus of this review is surveillance of mosquito-borne arboviruses of major public-health relevance, including dengue virus (DENV1–4), Zika virus (ZIKV), chikungunya virus (CHIKV), West Nile virus (WNV), Japanese encephalitis virus (JEV), yellow fever virus (YFV), and selected emerging mosquito-associated alphaviruses such as Mayaro virus. Within this scope, the evidence base is strongest for detection, whereas tracing and discovery-oriented applications still rely largely on platform demonstrations rather than integrated mosquito-borne virus surveillance deployments.

## 2 The CRISPR-Cas surveillance toolbox

CRISPR-Cas pathogen detection rests on three layers: a Cas effector that programmably recognizes a nucleic-acid target and triggers collateral cleavage; a pre-amplification step that lifts the target above the assay’s detection threshold in clinical or vector samples; and a readout that converts reporter cleavage into a result an operator can act on.

### 2.1 Cas effector families relevant to detection

CRISPR-Cas detection uses three Class 2 effector families with single-protein architectures whose target recognition is programmed by a small guide RNA: Cas12, Cas13 and Cas12f.

Cas12 (subfamilies Cas12a and Cas12b) recognizes double-stranded DNA targets and shows target-binding-triggered non-specific single-stranded DNase activity (Chen et al., 2018), a property that generalizes across the type V Cas12 family and is the basis of DETECTR-style assays. Cas13 (originally annotated as C2c2) is an RNA-guided RNA-targeting nuclease whose higher-eukaryotes-and-prokaryotes-nucleotide-binding (HEPN)-domain RNase activity triggers non-specific “collateral” cleavage of nearby single-stranded RNA on recognition of its guide-target complement (Abudayyeh et al., 2016; East-Seletsky et al., 2016). This converts a single pairing event into a multi-turnover signal-amplification step that underlies CRISPR-Cas RNA detection at attomolar sensitivity (Gootenberg et al., 2017). The Cas13a, Cas13b and Cas13d subfamilies share this mechanism but differ in protospacer-flanking site (PFS) preference and effector size. Cas13a is the most extensively used in arbovirus assays, Cas13d has been deployed in JEV detection, and Cas13b is included for taxonomic completeness. Cas12f, formerly referred to as Cas14, is a miniature CRISPR effector that cleaves single-stranded DNA targets without a protospacer-adjacent motif (PAM) requirement, broadening the detectable target set (Harrington et al., 2018).

Collateral cleavage shared between Cas12 and Cas13 is what makes CRISPR diagnostics mechanistically distinct from PCR. Rather than measuring amplicon concentration directly, the assay uses the amplicon as a trigger that liberates a downstream reporter signal, which adds a second layer of sequence specificity and removes the need for instruments that quantify amplicon concentration directly.

### 2.2 SHERLOCK, DETECTR, and the multiplex generation

SHERLOCK pairs isothermal recombinase polymerase amplification (RPA) with Cas13a collateral cleavage of fluorescent reporters. First validated on Zika and dengue virus targets, it achieves attomolar sensitivity with single-base-mismatch specificity (Gootenberg et al., 2017). SHERLOCKv2 extended this with four-channel single-reaction multiplexing using orthogonal Cas13 enzymes, input quantitation as low as 2 attomolar, and lateral-flow readout, converting SHERLOCK from a fluorescence-instrument assay into a strip-based field assay (Gootenberg et al., 2018). DETECTR provides the parallel platform on double-stranded DNA targets, using Cas12a’s single-stranded DNase activity with isothermal amplification (Chen et al., 2018), and is straightforwardly extended to RNA targets via a reverse-transcription step.

CARMEN redefined the multiplexing ceiling by using a microwell array to combinatorially pair sample droplets with crRNA droplets, enabling more than 4,500 crRNA-target pairings on a single array and a 169-pathogen multiplexed assay (Ackerman et al., 2020). The published platform was validated on cell-culture stocks and clinical specimens and requires custom microfluidic instrumentation. Portable, field-deployable CARMEN-style architectures for mosquito-pool homogenates remain an active engineering goal. With validated pooled-mosquito sample preparation and portable instrumentation, CARMEN-Cas13 could support targeted screening of regionally prioritized mosquito-borne arboviruses, particularly those associated with *Aedes* and *Culex* vectors, rather than functioning as an unbiased or exhaustive screen of the mosquito virome (Ackerman et al., 2020).

### 2.3 Pre-amplification and one-pot integration

Most CRISPR-Cas detection assays gain clinically usable sensitivity from a pre-amplification step that increases target concentration before the Cas reaction. RPA, loop-mediated isothermal amplification (LAMP), and the less commonly used Nicking Enzyme Amplification Reaction (NEAR) are the three dominant choices, each with trade-offs in primer-design complexity, reaction temperature and tolerance to non-specific priming.

The risk of a two-step amplification-then-detection workflow is aerosol contamination from amplicon escape, and the field response is one-pot integration. SHINE demonstrates the principle, where RPA-based amplification and Cas13-based detection occur in a single sealed tube with an in-tube fluorescent readout, validated on 50 nasopharyngeal samples with 90% sensitivity and 100% specificity at 50-minute time-to-result (Arizti-Sanz et al., 2020). The architecture generalizes across pathogen classes, with a 2023 one-pot RPA-CRISPR/Cas13a assay for Nipah virus and a 2022 one-pot LAMP-CRISPR system for *Klebsiella* showing its breadth of pathogen targets (Qiu et al., 2022; Miao et al., 2023), and a 2025 lyophilized extraction-free one-pot recombinase-aided amplification (RAA)-Cas13a device validated on bacterial clinical samples shows the engineering ceiling now reachable for ambient-temperature shipping (Cao et al., 2025).

### 2.4 Amplification-free CRISPR detection

Removing pre-amplification simplifies the workflow but sacrifices the orders-of-magnitude sensitivity gain that RPA and LAMP provide. The compensating engineering moves are signal-enhancing nanostructures and smartphone-based optical readouts. Direct mobile-phone microscopy of Cas13a reactions detects SARS-CoV-2 at amplification-free sensitivity sufficient for clinical use (Fozouni et al., 2021). The 2024 Solid-Phase Extraction and Enhanced Detection Assay integrated by CRISPR-Cas12a (SPEEDi-CRISPR) integrates target identification, sequence-specific magnetic-bead enrichment and on-bead fluorescence detection into a single workflow, dropping the limit of detection (LOD) from picomolar to femtomolar without pre-amplification and pairing with smartphone-readable fluorescence and lateral flow (Tian et al., 2024b). These amplification-free architectures have so far been validated primarily on SARS-CoV-2 rather than arboviruses. Translation to arbovirus targets remains an open engineering opportunity.

### 2.5 Readout architectures for point-of-need deployment

CRISPR-Cas readouts have diversified well beyond the original fluorescence-plate-reader format. In this review, point-of-care refers to testing performed at or near patient care, whereas point-of-need refers more broadly to testing performed near a surveillance or decision point, including peripheral laboratories, sentinel sites, ports of entry, mosquito-control programs, or trap-adjacent vector-surveillance settings. We use field-deployable only when an assay or workflow has been evaluated under field-relevant or field-use conditions; otherwise, we use deployment-oriented or field-compatible to describe formats designed for decentralized use but not yet field validated. Lateral-flow strips visualize Cas12/Cas13 collateral cleavage of dual-labelled reporters through accumulation of gold nanoparticles at a test line, the format used in deployment-oriented CRISPR-Cas dengue assay formats (Tian et al., 2024a). Smartphone-CMOS imaging substitutes a phone camera for the fluorescence reader, as demonstrated for amplification-free Cas13a SARS-CoV-2 detection (Fozouni et al., 2021). Label-free electrochemical biosensors, exemplified by the 2026 Cas13a-on-ceria CHIKV sensor, push the readout toward solid-state designs with multi-week stability (Zakiyyah et al., 2026).

## 3 Detection: rapid and variant-aware arbovirus diagnostics

Detection in mosquito-borne virus surveillance identifies a specific virus in a clinical or vector sample and, where assays are designed for it, resolves targeted serotype or lineage markers. In under a decade, the field has moved from first proof-of-concept demonstrations to clinically evaluated, assays and deployment-oriented formats for several major mosquito-borne arbovirus targets.

### 3.1 SHERLOCK assays for arboviruses

The 2017 SHERLOCK study used Zika and dengue virus as proof targets, demonstrating attomolar sensitivity and lyophilized reagents reconstituted on paper for use outside centralized laboratories (Gootenberg et al., 2017). The 2018 follow-up by Myhrvold and colleagues paired SHERLOCK with the Heating Unextracted Diagnostic Samples to Obliterate Nucleases (HUDSON) sample-lysis protocol to enable instrument-free detection directly from patient bodily fluids in under two hours, distinguishing the four DENV serotypes and assigning Zika strains to outbreak lineages from the 2015–2016 epidemic (Myhrvold et al., 2018). Together these studies showed that programmable nucleic-acid systems can support sensitive mosquito-borne virus detection in point-of-care-oriented formats. Subsequent work has shifted toward validation, workflow integration, and deployment engineering.

### 3.2 Per-pathogen CRISPR-Cas assays

Among the arboviruses, dengue remains the proving ground for CRISPR-Cas detection. Four antigenically distinct DENV serotypes co-circulate, clinical presentation overlaps with chikungunya, and serologic cross-reactivity with Zika and other co-circulating flaviviruses further complicates clinical management (Arif et al., 2026; Gularte et al., 2026). Among Cas12a-based designs, a one-pot RT-RPA + Cas12a configuration distinguishes all four DENV serotypes within 40 minutes, reaching ~92 copies per test (LOD) in fluorescence format and ~250 cp/test in lateral-flow dipstick (LFD) format, with no cross-reactivity against four interfering viruses (Zhang et al., 2025). Cas13a-based alternatives provide another approach, with a single-tube RAA + Cas13a configuration reaching ~10³ copies/mL at 95.8% average sensitivity and 96.6% specificity across the four DENV serotypes (Zhong et al., 2025). Dual cascades push sensitivity further, where a Cas13a-Cas12a architecture drops the LOD to 190 fM and yields naked-eye lateral-flow visualization (Tian et al., 2024a). Outside CRISPR-Cas, a 2025 RT-RPA + lateral-flow assay (DENV2-specific, without CRISPR coupling) provides an isothermal-amplification benchmark with ~50 copies-per-reaction LOD, robustness across 33–40 °C and repeated freeze-thaw cycles, and successful amplification from Flinders Technology Associates (FTA) cards (Prescott et al., 2025).

Zika-specific assays build on SHERLOCK. A 2026 RT-RPA + CRISPR/Cas12a configuration targeting the conserved capsid region achieves single-copy sensitivity, no cross-reactivity with DENV, CHIKV or JEV, and 100% concordance with RT-qPCR, with a naked-eye UV readout at 35 minutes that is compatible with point-of-care-oriented clinical screening, although broader field or peripheral-laboratory evaluation remains necessary (Cong et al., 2026). For chikungunya, three independent platform classes now exist: a 2024 RT-RPA + Cas12a one-pot dual-mode assay (~412 zg/μL, ≈ 1 copy; 100% sensitivity/specificity in undifferentiated febrile illness) (Bhardwaj et al., 2024); a 2026 SHERLOCK assay on 146 plasma samples (94.5%/100% lateral-flow, 97.3%/100% fluorescence) competitive with RT-qPCR for resource-limited outbreak response (Athipanyasilp et al., 2026); and a 2026 Cas13a-on-ceria electrochemical biosensor that detects CHIKV at 1.3 ppt with 45-day stability (Zakiyyah et al., 2026).

Single-target CRISPR-Cas designs target mosquito-borne or mosquito-associated arboviruses now in expanding or re-emerging ranges, including WNV in Europe, JEV in southern Australia and Southeast Asia, Getah virus in Asia, and Mayaro virus in the Americas. For WNV, a 2025 DETECTR variant uses RT-RPA + Cas12a to discriminate lineages 1a and 2 at ~10² RNA copies (Hwang et al., 2025). For JEV, a one-pot RT-RPA + Cas13d portable platform detects 2 copies in an hour with 100% concordance with RT-qPCR on clinical and vector samples, an architecture aligned with the One Health pig-farm surveillance practice that drives JEV epidemiology (You et al., 2024). For Getah virus, an RT-RPA + CRISPR/Cas12a + lateral-flow assay detects at 10 copies/μL in 50 minutes (Xia et al., 2025). For Mayaro virus, CRISPR-Cas evidence remains sparse, with a recently reported RT-RPA/CRISPR-Cas12a endpoint visual assay requiring broader clinical and vector-field validation (Marquez et al., 2026), consistent with the limited diagnostic evidence noted in recent emerging-threat reviews (Micheletti et al., 2026). **Table 1** summarizes per-pathogen primary studies.

**Table 1:** Evidence summary of CRISPR-Cas detection assays for mosquito-borne viruses.

Pathogen/target	Assay format	Cas/amplification	Sample/matrix	Validation size	LOD/analytical result	Readout/time	Evidence status	Reference
DENV strains and ZIKV strains	SHERLOCK platform	Cas13a + RPA	Synthetic targets; lentiviral particles; ZIKV-positive clinical serum or urine	Clinical sample count n.r.	Attomolar sensitivity; ZIKV patient serum/urine to 1.25 × 10³ copies/mL	Fluorescence; paper-based format shown	Foundational platform and clinical proof-of-concept; no field deployment	Gootenberg et al., 2017
ZIKV, DENV1–4, WNV, YFV targets; ZIKV SNPs	SHERLOCK + HUDSON	Cas13a + RPA	Patient cDNA; direct serum/saliva; ZIKV cDNA from pooled mosquito samples	ZIKV n = 16; DENV n = 24; direct DENV serum n = 8 and saliva n = 3	As low as 1 copy/μL; HUDSON matrix tests: 45 copies/mL in serum and about 1 copy/mL in saliva	Fluorescence, colorimetric, or LFD; <2 h	Clinical specimens plus cDNA from pooled mosquito samples; field-compatible workflow, not field trial	Myhrvold et al., 2018
Universal DENV1–4	One-pot RT-RPA-CRISPR/Cas12a; LFD adaptation	Cas12a + RT-RPA	Recombinant plasmids; DENV-infected cell samples; cultured DENV-2	22 DENV-infected cell samples plus 4 interference-virus samples; no clinical specimens	91.7 copies/test by fluorescence; about 250 copies/test by LFD	Fluorescence or LFD; 40 min	Analytical and cell-culture validation; no clinical/vector field validation	Zhang et al., 2025
DENV1–4	Single-tube RAA-CRISPR/Cas13a	Cas13a + RAA	Sample matrix not specified in abstract	n.r. in abstract	10³ copies/mL; average sensitivity 95.8% and specificity 96.6%	Fluorescence; time n.r.	Abstract reports diagnostic performance; sample set not detailed in abstract	Zhong et al., 2025
DENV	Cas13a-to-Cas12a dual-amplification cascade	Cas13a + Cas12a cascade	Analytical target material; matrix not specified in abstract	n.r. in abstract	190 fM by fluorescence; visible test line at 1 pM	Fluorescence and AuNP LFD; time n.r.	Abstract reports analytical platform demonstration; no clinical/vector field validation reported	Tian et al., 2024a
DENV serotypes and SARS-CoV-2	SHERLOCK with G4/hemin DNAzyme colorimetric readout	Cas13a + RPA	Cell-cultured DENV and DENV/SARS-CoV-2 clinical samples	n.r. in abstract	LOD n.r. in available abstract; accurate DENV serotype identification reported	Naked-eye colorimetric; time n.r.	Cell-culture and clinical proof-of-concept reported in abstract; no field validation reported	Zhang et al., 2026
ZIKV capsid gene	RT-RPA-CRISPR/Cas12a visual assay	Cas12a + RT-RPA	Clinical serum	39 serum samples: 5 positive and 34 negative	1 copy/μL; no cross-reactivity with DENV1–4, CHIKV, or JEV	UV visual fluorescence; 35 min	Clinical specimen validation with 100% concordance to RT-qPCR; no field deployment	Cong et al., 2026
CHIKV	One-pot RT-RPA-CRISPR/Cas12a dual-mode endpoint assay	Cas12a + RT-RPA	Clinical samples	n.r. in abstract	412 zg/μL, about 1 copy; 8 gene copies also reported	LFD and fluorescence; 35 min	Abstract reports clinical-sample validation with 100% sensitivity/specificity; sample count not detailed in abstract	Bhardwaj et al., 2024
CHIKV nsP1	SHERLOCK	Cas13a + RPA	Clinical plasma	146 plasma samples	215 copies/reaction; no cross-reactivity against tested alphaviruses or flaviviruses	LFD and fluorescence; 80 min including extraction	Clinical specimen validation: 94.52%/100% sensitivity/specificity by LFD and 97.26%/100% by fluorescence	Athipanyasilp et al., 2026
CHIKV RNA	Label-free electrochemical Cas13a-ceria SPCE biosensor	Cas13a; amplification-free	Spiked human serum	n.r. in abstract	1.325 ppt; recovery 94.98% in spiked serum	Differential pulse voltammetry; time n.r.	Spiked-sample analytical validation; no clinical/vector field validation reported in abstract	Zakiyyah et al., 2026
WNV lineage 1a and lineage 2	DETECTR: 2-step, 1-step, and 1-step-with-filter formats	Cas12a + RT-RPA	In vitro-transcribed RNA and WNV lenti-pseudovirus RNA	No clinical samples; sequence coverage predicted from available WNV sequences	1.0 × 10² RNA copies/reaction for 2-step and filter formats; 1.0 × 10³ for 1-step	Fluorescence or LFA; RT-RPA 40 min plus Cas12a readout	Analytical and pseudovirus validation; clinical samples still required	Hwang et al., 2025
Swine JEV	One-pot RPA-CRISPR/EsCas13d portable platform	EsCas13d + RPA	Clinical samples	n.r. in abstract	2 copies; no cross-reactivity with other pathogens	Fluorescence and LFA; 1 h	Abstract reports clinical-sample concordance with real-time PCR; sample count not detailed in abstract; genotype discrimination not inferred	You et al., 2024
Getah virus E2 gene	RT-RPA-CRISPR/Cas12a with LFD or fluorescence	Cas12a + RT-RPA	Porcine serum simulated clinical samples	36 simulated samples, including 6 GETV-positive serum-spiked samples	10 copies/μL; no cross-reactivity with tested veterinary viruses	LFD or fluorescence; 50 min	Simulated clinical validation with 100% concordance to RT-qPCR; no true-positive clinical validation	Xia et al., 2025

CHIKV, chikungunya virus; DENV, dengue virus; GETV, Getah virus; JEV, Japanese encephalitis virus; WNV, West Nile virus; YFV, yellow fever virus; ZIKV, Zika virus; AuNP, gold nanoparticle; LFA, lateral-flow assay; LFD, lateral-flow detection; LOD, limit of detection; n.r., not reported; RAA, recombinase-aided amplification; RPA, recombinase polymerase amplification; RT-RPA, reverse-transcription recombinase polymerase amplification; RT-qPCR, reverse-transcription quantitative PCR; SPCE, screen-printed carbon electrode.

### 3.3 Variant-aware detection

CRISPR-Cas guide-RNA design can support discrimination of predefined single-nucleotide mismatches, the property that gives SHERLOCK its reported single-base specificity under designed assay conditions (Gootenberg et al., 2017). Programmable variant-aware assays can therefore distinguish selected serotype, genotype, lineage or adaptive substitution markers when those markers are known in advance and incorporated into the guide design. This targeted discrimination should be distinguished from genome-wide lineage reconstruction, which requires sequencing. WNV exemplifies this, where lineage 1a predominates in the Americas and southern Europe while lineage 2 has expanded across central and northern Europe over the past two decades, making lineage-resolved detection relevant for European surveillance (Koch et al., 2024; Vignjević et al., 2024), and the 2025 DETECTR variant discriminates lineage 1a from lineage 2 at ~10² RNA copies (Hwang et al., 2025). Below this lineage-level split, recent genomic-epidemiology work in Croatia and France defines the sub-lineage structure that future crRNA designs can target (Anđelić Dmitrović et al., 2025; Klitting et al., 2026).

For dengue, the priority is serotype-resolved detection. In silico-designed Cas13 guide RNAs target conserved DENV genomic regions, including NS5 and non-coding viral genomic regions reported in the guide-design study, alongside serotype-specific variable regions, providing the bioinformatic foundation for DENV1–4 discrimination (Prajapati et al., 2022), and the one-pot RT-RPA + Cas12a + lateral-flow assay implements this design as a 40-minute field assay that distinguishes all four serotypes (Zhang et al., 2025). Below the serotype level, regional genomic surveillance routinely confronts lineage-level questions (Carvalho Marques et al., 2023; Perez et al., 2025). A recent lineage-nomenclature framework now categorizes sub-serotype and major lineages for variant-aware crRNA design (Hill et al., 2024). For chikungunya, CRISPR-Cas can match the variant-aware precedent set by nested RT-PCR assays for discriminating East/Central/South African (ECSA) versus Asian lineages and E1/E2-region adaptive substitutions broadly across CHIKV genotypes (Fischer et al., 2019). For Zika, recent phylogenomic analyses provide the lineage map underlying variant-aware CRISPR-Cas design (Zadra et al., 2024). For JEV, the RT-RPA + Cas13d portable platform provides sensitive JEV detection in a One Health swine-surveillance context, but genotype-discriminating performance has not been established from the available evidence (You et al., 2024). **Table 2** summarizes these per-pathogen assays.

**Table 2:** Variant-aware CRISPR-Cas assays and target-design precedents for mosquito-borne viruses.

Pathogen/lineage	Target region	Guide strategy	Resolution	LOD/concordance	Notes	Reference
WNV lineage 1a vs lineage 2	C gene for lineage 1a; M-E non-coding region for lineage 2	RT-RPA + Cas12a with lineage-distinguishing crRNAs	Lineage 1a vs lineage 2	~10² RNA copies; reported sensitivity similar to qRT-PCR	DETECTR-based WNV lineage assay; relevant to WNV lineage 2 expansion in Europe	Hwang et al., 2025
DENV1–4	Viral non-coding region and NS5 conserved regions plus NS2A/NS2B variable regions	In silico-designed Cas13a guides; one effector with four serotype-specific guides	DENV serotype 1 vs 2 vs 3 vs 4	Bioinformatic guide-design study; no experimental LOD	Design foundation for serotype-resolved CRISPR-Cas assays; not counted as experimental validation in **Table 1**	Prajapati et al., 2022
DENV1–4	Serotype-specific NS regions	RT-RPA + Cas12a with fluorescence or LFD readout	DENV serotype 1 vs 2 vs 3 vs 4	~92 copies/test by fluorescence; ~250 copies/test by LFD; no cross-reactivity reported	40-min one-pot dual-readout assay for DENV serotype discrimination	Zhang et al., 2025
CHIKV ECSA, Asian, and Indian Ocean lineages	E1 and E2 envelope genes	Nested RT-PCR lineage-discrimination precedent; design template for future CRISPR-Cas guide development	Genotype and selected vector-adaptation mutations	Comparable to standard RT-PCR in the cited molecular-surveillance precedent; no CRISPR-Cas LOD	Non-CRISPR nested RT-PCR precedent; included only as target-design precedent for future CHIKV variant-aware CRISPR-Cas assays	Fischer et al., 2019
ZIKV Asian and African lineages	Whole-genome lineage-discriminating positions	Phylogenomic lineage framework for future target selection	Lineage and sublineage discrimination as an in silico foundation	n.r.; phylogenomic foundation rather than diagnostic validation	Not a CRISPR guide-design study; CRISPR-Cas lineage assay remains pending	Zadra et al., 2024
Swine JEV	JEV nucleic-acid target; genotype coverage not established from available evidence	One-pot RPA + Cas13d portable platform	JEV detection; not genotype discrimination	2 copies; reported 100% concordance with RT-qPCR	Dual fluorescence and LFD readout; aligned with One Health swine/JEV surveillance use case; do not describe as genotype-aware without full-text confirmation	You et al., 2024

CHIKV, chikungunya virus; DENV, dengue virus; ECSA, East/Central/South African lineage; JEV, Japanese encephalitis virus; LFD, lateral-flow detection; LOD, limit of detection; n.r., not reported; RPA, recombinase polymerase amplification; RT-RPA, reverse-transcription recombinase polymerase amplification; WNV, West Nile virus; ZIKV, Zika virus.

### 3.4 Engineering point-of-need deployment

For point-of-need mosquito-borne virus detection, the Cas reaction is only one part of the translational bottleneck. Upstream sample preparation, reagent stability, and end-to-end validation remain central. The foundational SHERLOCK and HUDSON work demonstrated that Cas13a reagents can be lyophilized for cold-chain independence and reconstituted on paper, with an extraction-free heat-lysis protocol carrying detection directly from patient bodily fluids (Gootenberg et al., 2017; Myhrvold et al., 2018). A 2025 lyophilized extraction-free one-pot RAA-Cas13a device (EFORCA) integrates these elements in a bacterial assay and ships at ambient temperature, illustrating a platform architecture that still requires mosquito-borne virus-specific translation and validation (Cao et al., 2025). Solid-phase Cas12a enrichment-and-detection designs reach femtomolar limits without pre-amplification (Tian et al., 2024b), and a colorimetric SHERLOCK using a guanine-quadruplex/hemin DNAzyme cascade detects DENV by naked-eye color change, reducing reliance on optical instruments (Zhang et al., 2026).

For decentralized deployment, the principal comparator is often reverse-transcription LAMP (RT-LAMP) rather than RT-qPCR. Selected CRISPR-Cas assays report attomolar to femtomolar analytical limits of detection, 30–60 min workflows, lateral-flow or fluorescence readouts, and multi-target formats, but these performance claims remain assay-, target-, and sample-matrix-dependent. Cost estimates of roughly USD 1–5 per test are largely extrapolated from non-arbovirus or platform-level reports rather than field evaluations of mosquito-borne virus assays. CRISPR-Cas retains a multiplexing advantage through orthogonal effectors and CARMEN-style microwell architectures, but multiplexing, sample preparation, cold-chain tolerance, and readout interpretation still require target-specific validation (Gootenberg et al., 2018; Ackerman et al., 2020). By contrast, RT-LAMP offers lower cost for single-target deployment, a longer field-validation track record for several arbovirus targets, and higher tolerance to inhibitors in some unprocessed samples (Arkell et al., 2023). Taken together, these complementary profiles suggest a tiered architecture in which LAMP coupled to lateral-flow assay (LAMP-LFA) handles peripheral single-target screening and CRISPR-Cas handles serotype/lineage discrimination or multiplex screening when validation supports its use. **Table 3** summarizes the practical trade-offs among RT-qPCR, RT-LAMP/LAMP-LFA, CRISPR-Cas detection, amplicon sequencing, metagenomic NGS, and CARMEN-style targeted multiplex screening for mosquito-borne virus surveillance.

**Table 3:** Practical comparison of molecular tools for mosquito-borne virus surveillance.

Method	Best-fit use case	Sample preparation/cost profile	Multiplexing	Validation maturity	Regulatory maturity	Main limitation
RT-qPCR	Central or regional laboratory confirmation and quantification of known targets	Usually requires RNA extraction, cold-chain-compatible reagents, and laboratory instrumentation; moderate-to-high per-run infrastructure cost	Low to moderate; limited by fluorophore channels and primer/probe interactions	High for many mosquito-borne virus targets; widely used as reference molecular method	Most mature among the molecular methods compared here	Requires real-time PCR instrumentation and centralized laboratory capacity
RT-LAMP/LAMP-LFA	Low-cost point-of-need screening when one or a few targets dominate	Simpler heating equipment and potentially lower per-test cost; extraction or heat-treatment requirements vary by assay	Usually low to moderate	Longer field-use and field-evaluation record than CRISPR-Cas for several arbovirus targets	Variable by assay and jurisdiction; generally more established than CRISPR-Cas for field screening	Primer design is complex; nonspecific amplification and limited multiplexing can complicate interpretation
CRISPR-Cas detection	Targeted detection with serotype-, genotype-, or lineage-aware discrimination	Often requires RT-RPA, RT-LAMP, RAA, or PCR pre-amplification; reagent cost estimates remain mostly platform-derived rather than field-validated for mosquito-borne virus assays	Moderate; orthogonal effectors allow multi-channel detection	Technically promising but uneven across mosquito-borne virus targets and sample matrices	Regulatory clearance for mosquito-borne virus targets was not identified in the reviewed literature	Field performance depends on amplification compatibility, sample preparation, inhibitor tolerance, and target-specific validation
Amplicon sequencing	Outbreak tracing, lineage reconstruction, and phylodynamic analysis when the target virus is known	Requires RNA extraction, reverse transcription, multiplex PCR, library preparation, sequencing consumables, and bioinformatics	Genome-wide within the targeted primer scheme	Established in multiple arbovirus outbreak investigations	Used in public-health genomics, but regulatory maturity is workflow- and setting-dependent	Primer mismatch, low viral load, and sequencing infrastructure can limit recovery
Metagenomic NGS	Unbiased detection of divergent or unexpected viruses and characterization of vector viromes	Requires RNA extraction and often host/rRNA depletion, enrichment, or deep sequencing; generally higher cost and bioinformatics burden	Broad and untargeted	Strong discovery value but operationally demanding for routine surveillance	Limited regulatory maturity for routine diagnostics	Lower sensitivity for low-titer targets without enrichment; higher cost and bioinformatics burden
CARMEN-style targeted multiplex screening	High-plex screening of known or near-neighbor targets covered by predesigned crRNAs	Requires compatible sample preparation, amplification, predesigned crRNAs, and platform-specific instrumentation; potential reagent savings depend on scale and platform access	Very high for represented targets	Platform-scale promise; limited validation in pooled mosquito samples	Not established for routine mosquito-borne virus surveillance	Cannot discover targets outside the panel; requires portable instrumentation and vector-sample validation

CARMEN, Combinatorial Arrayed Reactions for Multiplexed Evaluation of Nucleic acids; LAMP-LFA, loop-mediated isothermal amplification coupled to lateral-flow assay; NGS, next-generation sequencing; RAA, recombinase-aided amplification; RPA, recombinase polymerase amplification; RT-LAMP, reverse-transcription loop-mediated isothermal amplification; RT-qPCR, reverse-transcription quantitative PCR.

### 3.5 Interpreting pooled mosquito samples

Large-scale mosquito-borne virus surveillance usually tests pooled mosquitoes rather than individual insects. A mosquito pool is typically assembled by trap, date, location, species, and sometimes sex or physiological status, and then homogenized for nucleic-acid extraction or direct testing. A positive pooled sample therefore indicates viral RNA in that pool, not an individually infected mosquito. This format is established in WNV programs and metatranscriptomic surveillance, where pooled samples are interpreted together with species identification, collection metadata, and confirmatory RT-qPCR or sequencing (Bernard et al., 2001; Engler et al., 2013; Batovska et al., 2022). This distinction is important for CRISPR-Cas assays because analytical sensitivity measured with purified RNA, plasmids, cultured virus, or clinical specimens does not automatically translate to pooled mosquito homogenates.

Pre-analytical variables should therefore be reported explicitly in CRISPR-Cas vector-sample studies. These include pool size, species identification method, whether pools are species-resolved or mixed-species, sex and physiological status where relevant, storage conditions from trap to extraction, preservation medium, homogenate volume, extraction method, and the presence or removal of mosquito-derived inhibitors. Larger pools improve throughput but dilute low-abundance viral RNA and increase the amount of host, microbial, and environmental material in the homogenate. Poor cold-chain control, repeated freeze-thaw cycles, or delayed processing can accelerate RNA degradation, whereas blood-fed, gravid, or sugar-fed mosquitoes may differ in inhibitor burden and background nucleic-acid composition. These variables determine whether a CRISPR-Cas assay developed on clean templates remains usable in operational vector surveillance.

Pooling also changes how positive results are interpreted epidemiologically. Minimum infection rate (MIR) assumes at least one infected mosquito in each positive pool and expresses positives per number of mosquitoes tested, whereas maximum likelihood estimation (MLE) uses pool-size information to estimate infection prevalence. Both metrics depend on pool design, retention of original pools, and retesting strategy, and pooled RT-PCR studies show that infection-rate estimates are most informative when original pools are retained for deconvolution or when pool-size information is preserved (Condotta et al., 2004; Chisenhall et al., 2008). CRISPR-Cas vector-sample studies should therefore report positive-pool counts together with pool sizes and MIR or MLE where the sampling design permits, and should benchmark pooled-sample performance against RT-qPCR or metatranscriptomic sequencing rather than relying only on clinical-sample LOD.

Finally, detection of viral RNA in mosquitoes or mosquito pools should be distinguished from vector competence and vectorial capacity. Field detection indicates that viral RNA was present in an individual mosquito or pooled sample at the time of collection, but it does not establish that the mosquito supported productive infection or could transmit infectious virus. Vector competence is usually assessed experimentally through sequential endpoints: infection of the mosquito body or midgut, dissemination beyond the midgut to secondary tissues, and transmission potential demonstrated by infectious virus in saliva (Wu et al., 2025). Vectorial capacity is broader still, incorporating vector density, biting rate, survival, host preference, extrinsic incubation period, and ecological context in addition to competence. For CRISPR-Cas surveillance, a positive mosquito pool should therefore be interpreted as a trigger for follow-up testing, species confirmation, spatial clustering analysis, sequencing, virus isolation, or vector-control action, rather than as direct evidence that the sampled population is a competent vector or that local transmission is occurring.

## 4 Tracing: real-time outbreak phylodynamics

Whereas detection answers what is here, tracing reconstructs where the virus came from and where it is going from recovered pathogen sequences. In mosquito-borne virus surveillance, the validated outbreak-tracing evidence currently comes mainly from portable amplicon-sequencing workflows, especially ARTIC/ZiBRA-style tiling-PCR approaches. CRISPR-aided methods such as DASH, FLASH, RAPID-DASH, and Cas9-targeted nanopore enrichment are therefore discussed here as sample-preparation strategies that could improve host depletion or target enrichment, rather than as workflows already integrated into mosquito-borne virus genomic surveillance. As outbreaks accelerate, sample-to-phylogeny turnaround remains a limiting variable for response, and sample preparation is often a major contributor to that turnaround.

### 4.1 CRISPR-aided sample preparation as a transferable opportunity

The main bottleneck for viral genomic surveillance from clinical and vector samples is signal-to-noise, since low-titer mosquito-borne viruses are often diluted in excess host or vector nucleic acid. CRISPR-Cas methods could address this bottleneck at the library-preparation step by depleting abundant non-target sequences or enriching targets of interest, but most evidence for these methods still comes from non-arbovirus or non-integrated sequencing contexts. DASH, the foundational method, uses Cas9 ribonucleoproteins to remove high-abundance reads — initially mitochondrial rRNA — before final amplification (Gu et al., 2016). Subsequent work has extended DASH to bacterial RNA-seq, single-cell formats, small-RNA sequencing, long-read hemoglobin depletion (Byrne et al., 2019; Hardigan et al., 2019; Prezza et al., 2020; Loi et al., 2021), and to low-viraemia specimens via a nanopore protocol combining sequence-independent single-primer amplification (SISPA) with DASH (Selhorst et al., 2026).

Whereas DASH depletes abundant non-target reads, FLASH uses Cas9 to positively select target sequences, achieving up to five orders of magnitude of enrichment and sub-attomolar gene detection in its original antimicrobial-resistance application (Quan et al., 2019). For mosquito-borne virus tracing, FLASH should therefore be viewed as a transferable enrichment strategy rather than a validated arbovirus surveillance workflow. Its use in mosquito pools or early-acute-phase clinical samples would require mosquito-borne virus-specific guide design, benchmarking against amplicon sequencing or metagenomic NGS, and validation across relevant sample matrices. Other pathogen-specific deployments include a 2022 *Legionella pneumophila* FLASH application recovering whole-genome phylogenetic resolution without culture (Domazetovska et al., 2022), and a 2025 *Klebsiella pneumoniae* workflow combining Cas9 enrichment with Oxford Nanopore sequencing (Cottingham et al., 2025).

Recent work multiplexes the gRNA array itself. RAPID-DASH assembles gRNA arrays of up to 10 units in a day, enabling combinatorial depletion or enrichment that could scale to field surveillance (Salaudeen et al., 2026). Beyond library-level enrichment, Cas9 ribonucleoproteins can direct cleavage upstream of a target locus during nanopore sequencing itself, producing PCR-free targeted reads benchmarked at 643- and 468-fold enrichment in forensic short-tandem-repeat profiling (Yang et al., 2026), though the authors concluded that Cas9-seq was less favorable than alternative approaches for STR profiling specifically. Outside CRISPR-Cas, FP-NSA pairs multiplex family-wide PCR with Nanopore amplicon sequencing, screening influenza and coronavirus samples from PCR to interpretable reads in 4 hours (Meki et al., 2025). Taken together, these CRISPR-aided approaches are best interpreted as library-preparation or enrichment modules that may improve future mosquito-borne virus sequencing workflows. They should not yet be treated as substitutes for validated outbreak-sequencing pipelines until they are integrated with arbovirus primer schemes or metagenomic workflows and evaluated on clinical and pooled-vector samples.

### 4.2 Validated arbovirus outbreak-sequencing workflows

By contrast, the strongest validated evidence for real-time arbovirus tracing comes from amplicon-sequencing workflows. The ARTIC Network multiplex tiling-PCR scheme on the portable MinION platform has recovered near-complete viral genomes from clinical samples in 1–2 days in optimized workflows, with reported sensitivity down to 50 genome copies for Zika and related viral targets (Quick et al., 2017). In 2016, the ZiBRA project ran a mobile sequencing laboratory across northeast Brazil, sharing molecular diagnostic results with state public-health laboratories within 48 hours of analysis, and the molecular-clock analysis of the 54 ZIKV genomes indicated that ZIKV was already present in northeast Brazil by February 2014, more than a year before its first formal detection (Faria et al., 2017). Subsequent studies extended the ARTIC scheme to Zika in Colombia (Black et al., 2019), the 2016–2019 yellow fever resurgence in southeast Brazil (Faria et al., 2018; Giovanetti et al., 2019), and the post-Zika Brazilian dengue resurgence (Brito et al., 2021).

Extending to chikungunya, dengue and mosquito surveillance, the same scheme has been used across Latin America, Asia, Africa and Oceania. For chikungunya, retrospective genomic surveillance in Minas Gerais identified three independent ECSA-lineage CHIKV introductions into the state between 2017 and 2021, likely from the North and Northeast regions of Brazil (Fritsch et al., 2022). For dengue, mainland China studies have tracked imported-case seeding across tropical, subtropical and temperate zones (Wu et al., 2022), while a 2023 Guangzhou outbreak study integrated 2010–2019 epidemiological and genomic data (Jiang et al., 2023). Outside Asia, a 2024 Benin study first detected DENV-1 and DENV-3 (Yadouleton et al., 2024). Similarly, the pipeline is applied for mosquito surveillance. A 2022 Australian study sequenced 56,000 trapped mosquitoes following a 2016 flood event in Victoria and detected five public-health-relevant arboviruses 33 times across seven weeks (Batovska et al., 2022), mosquito-excreta metagenomics offers a less-invasive sampling alternative (Ramírez et al., 2020), and a Kenyan vertical-transmission study combined cell culture with metagenomic sequencing on 86 *Aedes aegypti* pools to detect DENV-3 with evidence of transovarial transmission (Wanjiru et al., 2025).

Sequencing capacity disparities across regions remain a major gap, as a recent dengue genomic surveillance review identifies infrastructure and sequencing-capacity barriers (Alcantara et al., 2026). CRISPR-aided sample preparation could help close part of this gap by improving host or vector nucleic-acid depletion and pathogen-targeted enrichment, thereby reducing sequencing depth and downstream bioinformatic burden. However, this remains a translational opportunity for mosquito-borne virus surveillance rather than an established outbreak-tracing workflow, and it will require integrated demonstrations with clinical and pooled-vector samples before peripheral deployment can be assessed.

## 5 Discovery: targeted multiplex screening for emerging mosquito-borne viruses

Whereas detection and tracing work on defined pathogens and recovered genomes, discovery-oriented surveillance combines targeted screening with unbiased sequencing. In this review, CRISPR-Cas contributes to this function by prioritizing known or near-neighbor mosquito-borne virus targets through multiplex assays, while metagenomic NGS remains necessary for highly divergent or previously unknown viruses. As emergence cycles compress, multiplex CRISPR-Cas screening may help triage represented targets rapidly, but it does not replace metagenomic sequencing as the discovery step for targets outside the panel.

### 5.1 Re-emerging and previously-overlooked arboviruses

Arbovirus emergence and re-emergence have accelerated over recent decades in patterns targeted assays miss by design. Experimental studies indicate that some populations of *Aedes japonicus japonicus* can support Zika virus transmission under specific temperature conditions, suggesting a possible temperate-zone risk window rather than establishing uniform vector competence across this species (Glavinic et al., 2020). Existing sublineages are spreading, with West Nile virus lineage 2 sublineage emergence across Europe motivating revisiting WNV as a sentinel for arboviral surveillance change (Koch et al., 2024; Vignjević et al., 2024). Long-archived arboviruses are being re-evaluated, with systematic re-examination of viruses discovered in the 1950s–1980s reshaping the catalogue of potentially re-emergent ones (Claro et al., 2025). And new outbreaks come on shorter cycles, with recent updates documenting the continued expansion or re-emergence of mosquito-associated arboviruses such as Mayaro virus (Micheletti et al., 2026). Together, these patterns show why surveillance systems need assays that can be updated as mosquito-associated arbovirus threats shift across regions and transmission seasons.

### 5.2 Insect-specific viruses as surveillance context

Mosquitoes carry far more viral diversity than the arboviruses that cause human disease, with insect-specific viruses (ISVs), which replicate only in the vector, making up the bulk and acting as both a methodological challenge that crowds out arboviruses in untargeted sequencing and a biological signal whose composition changes vector competence (Bolling et al., 2015; Altinli et al., 2021).

Recent atlases draw an unexpectedly heterogeneous picture. A 2023 global database from 175 metagenomic studies shows that mosquito-virome composition varies between mosquito species and biogeographic regions, with each species carrying its own distinct core rather than a universally shared one (Moonen et al., 2023). Consistent with this, temperate *Culex* populations in Belgium lack an abundant core virome (De Coninck et al., 2024), *Aedes* in southwestern Cameroon show no core but rich ISV diversity (Mbigha Donfack et al., 2024), and large-scale surveys link virome composition to mosquito species and environment rather than a conserved core (Liu et al., 2023a).

ISVs are more than methodological background. Through co-infection, they shape arbovirus replication via shared RNAi machinery and resource competition (Altinli et al., 2022; Shi et al., 2022; Agboli et al., 2023; Jansen et al., 2025), and their diversity serves as a population-genetic readout for mosquito structure (Hollingsworth et al., 2023). ISVs therefore set both the noise floor against which targeted screening or metagenomic sequencing must perform and the biological context for interpreting new or unexpected viral signals.

### 5.3 Multiplex CARMEN-Cas13 for discovery-supporting targeted screening

Where metagenomic NGS provides untargeted coverage, CRISPR-Cas multiplex assays provide targeted coverage, with pre-programmed crRNAs against conserved regions of selected viral groups turning a single sample into a multi-target screen. CARMEN demonstrates the principle at scale, with a microwell array that combinatorially pairs sample droplets with crRNA droplets, enabling >4,500 crRNA-target pairings on a single array and a 169-pathogen multiplexed assay (Ackerman et al., 2020). Recent extensions include a 2025 multiplexed CARMEN deployment for febrile-infection differential diagnosis, validated on clinical samples and providing a template for vector-pool adaptation (Kamariza et al., 2025), and a 2023 Cas13 mRNA-quantitation preprint demonstrating scalable RNA quantitation comparable to gold-standard methods. Beyond multi-pathogen multiplexing, the same architecture supports lineage-resolution screening at scale, with the WNV lineage 1a vs 2 DETECTR variant providing a working precedent (Hwang et al., 2025).

While the published CARMEN platform was validated on cell-culture stocks and clinical specimens with custom microfluidic instrumentation, field-portable instruments and validated mosquito-pool sample-prep workflows remain to be engineered. This engineering frontier is catalogued by recent reviews of microfluidic technologies for field-portable multiplex platforms (Wang et al., 2024) and of CRISPR-Cas integration with Argonaute nucleases identifying multiplex capability as a key development area (Xu and Wu, 2026). CRISPR multiplex screening can provide a targeted first pass for viruses represented in the predesigned crRNAs, with metagenomic NGS retained as the unbiased method for targets not covered by those crRNAs. Together, this two-stage workflow could trigger expected or near-neighbor signals quickly while preserving sequencing as the essential discovery step for divergent or unknown viruses. Pool-based metagenomic platforms show this NGS fallback scaling to vector pools, with a Senegal study extending detection to retroviruses alongside arboviruses (Ndione et al., 2025) and SMART-RNA-Metavirome batch-processing vector-pool RNA at routine-surveillance throughput (Liu et al., 2025).

## 6 Discussion

Across the three CRISPR-Cas functions reviewed, the evidence base for mosquito-borne virus surveillance is uneven. Detection is the most advanced application. Selected one-pot assays report sub-hour workflows, analytical performance that can approach RT-qPCR under defined assay conditions, and portable readouts, but validation depth and field evidence differ across mosquito-borne virus targets and sample matrices. Serotype- or lineage-aware discrimination is increasingly feasible within targeted detection workflows. Tracing has proven sequencing platforms but lacks integrated CRISPR demonstrations. The ARTIC scheme is established in arbovirus outbreaks across Brazil, Asia, Africa, and Oceania (Faria et al., 2017; Quick et al., 2017). CRISPR-aided sample-preparation methods, including DASH, FLASH, and RAPID-DASH, have been validated mainly in bacterial or non-arbovirus contexts (Quan et al., 2019), but have not yet been integrated into mosquito-borne virus genomic surveillance. Discovery-supporting targeted multiplex screening has platform-scale promise, since CARMEN can cover thousands of crRNA-target pairings on cell-culture and clinical specimens (Ackerman et al., 2020). However, this remains targeted screening rather than unbiased discovery, and field-portable instrumentation, pooled-mosquito sample preparation, low-abundance target performance, and validation against metagenomic sequencing remain unresolved. Across the three functions, routine surveillance readiness must be assessed function by function.

What makes CRISPR-Cas structurally different from RT-qPCR, RT-LAMP and amplicon-resequencing alternatives is not that retargeting is reduced to guide exchange alone, but that guide redesign can be separated from other parts of the assay stack. RT-qPCR and RT-LAMP retarget primer sets whose validation cycles can be longer than guide redesign, while amplicon-resequencing schemes such as ARTIC tiling PCR require primer performance across each target window (Quick et al., 2017). Most CRISPR-Cas diagnostic assays still require compatible RT-RPA, RT-LAMP, or PCR primers, effector-specific target constraints such as PAM requirements for Cas12 assays or PFS preferences for some Cas13 assays, and empirical cross-reactivity and analytical validation. The practical advantage is largest when conserved amplification primers can be retained and only the crRNA is changed, or when predesigned guide sets can be expanded without rebuilding the full assay (Huang et al., 2024). This programmability can scale from single-reaction multiplexing through orthogonal Cas effectors (Gootenberg et al., 2018) to CARMEN-class screening on microwell arrays (Ackerman et al., 2020). As DENV serotypes, WNV lineage 2 sublineages, CHIKV ECSA sub-clades, and other mosquito-borne virus lineages change and co-circulate, these properties remain practical surveillance advantages, but they do not remove the need for target selection, amplification design, and assay validation.

These advances are bounded by several limitations. First, trans-cleavage gives Cas12/Cas13 their signal-amplification advantage but also leaves multiplexed reactions vulnerable to interference between adjacent reporters when scaling beyond the 4-channel SHERLOCKv2 architecture toward CARMEN-scale multiplexing. Second, DENV, ZIKV, WNV, JEV and YFV share substantial sequence conservation across NS5 and capsid regions, so broad-coverage guide design becomes a liability for differential diagnosis when multiple flaviviruses co-circulate. Third, lyophilized one-pot reagents have largely solved the cold-chain problem at the assay step but only partially address reagent failure at temperatures above 35 °C and humidity above 80%, and extraction-free sample preparation directly from blood or mosquito pools has not been engineered for arbovirus targets. Fourth and most consequentially, CRISPR-aided enrichment and CARMEN-class multiplex screening have not yet been demonstrated during arbovirus outbreaks, and variant-aware CRISPR assays have published validation primarily in retrospective and small prospective studies. Beyond these technical concerns, the USD 1–5 per-test cost figure is extrapolated from platform reports rather than backed by mosquito-borne virus-specific field data, and no CRISPR-Cas assay has yet been FDA-cleared or WHO-prequalified for any mosquito-borne virus target. The SARS-CoV-2 DETECTR Emergency Use Authorization (EUA) precedent illustrates a possible regulatory route, but it does not substitute for arbovirus-specific regulatory evidence. The 2026 molecular-diagnostics review names global standardization, regulatory pathways and infrastructure as the key remaining barriers (Asokan et al., 2026).

Within these limitations, potential deployment pathways align with existing mosquito-control programs. *Wolbachia* release co-monitoring is one path. Releases of wMel- or wAlbB-infected *Aedes* mosquitoes in dengue-endemic regions require monitoring of release fidelity, *Wolbachia* strain persistence, vector population structure, and arbovirus circulation (Edenborough et al., 2024; Paz-Bailey et al., 2025; Lim et al., 2026). However, the fraction of trapped *Aedes* carrying an introduced *Wolbachia* strain should not be inferred from an undifferentiated mosquito-pool homogenate. Individual mosquito testing remains the most direct approach when fine-scale release fidelity or infection frequency is the endpoint. Pooled testing can support monitoring only when the sampling scheme is designed for pooled-prevalence estimation, with known pool sizes, species-resolved pools, retention of original pools for deconvolution where possible, and appropriate statistical estimation. In this setting, a multiplex CRISPR-Cas assay could serve as a screening layer for *Wolbachia* strain markers and arbovirus RNA in individual mosquitoes or statistically designed pooled samples, with positive pooled results routed to deconvolution, RT-qPCR confirmation, sequencing, or targeted follow-up rather than interpreted as direct individual-level frequencies (Condotta et al., 2004; Chisenhall et al., 2008; Turner et al., 2023). Smart-trap-coupled detection is another path. Internet-of-Things mosquito traps with on-device species classification (Liu et al., 2023b; Njaime et al., 2024; González-Pérez et al., 2026) produce species-resolved mosquito pools suitable for on-trap or trap-adjacent multiplex CRISPR detection with hits routed to portable sequencing. Singapore’s five-decade dengue prevention program (Ho et al., 2023) and integrated-control arguments (Côrtes et al., 2023) provide a working model for embedding multiplex detection alongside vaccination and vector control. Beyond these existing paths, future directions include but are not limited to:

1. Field-scale multiplex detection. Laboratory CARMEN-style microwell arrays already support >4,500 crRNA-target pairs at >300-fold reagent-cost reduction per test (Ackerman et al., 2020), and one next step is field-portable microwell or microfluidic platforms that screen regionally prioritized mosquito-borne virus targets in species-resolved or statistically designed pooled mosquito samples.
2. AI-aided design and smartphone readouts. On the design side, deep-learning-guided crRNA design improves on-target prediction for Cas12a-based detection (Huang et al., 2024). On the readout side, smartphone-image classification on lateral-flow strips and microwell plates moves readout from benchtop instruments to hardware operators already carry, standardizing interpretation across operators with varied training.
3. Variant-responsive surveillance. Mosquito-borne virus lineages and serotypes can change faster than legacy assay-redesign workflows. CRISPR-Cas systems can be reprogrammed on guide-design and synthesis timescales, but full assay retargeting still requires compatible primers, cross-reactivity checks, and analytical validation. Variant-aware assay designs and bioinformatic pipelines such as PrimedSherlock can streamline crRNA and primer design for emerging variants (Mann and Pitts, 2022).

The CRISPR-Cas platforms covered in this review define a technically promising toolkit for mosquito-borne virus surveillance, with detection the closest to operational evaluation. Integrated demonstrations during mosquito-borne virus outbreaks, pooled-vector-sample workflows, comparative validation against established molecular and sequencing methods, and regulatory pathways are needed before routine surveillance use can be judged across functions. As mosquito-borne virus serotypes and lineages continue to change and co-circulate, the cost-of-redesign and multiplex advantages of CRISPR-Cas warrant careful evaluation rather than assumptions of routine readiness.
